# Future time perspective and its association with employment anxiety among college students: a moderated mediation model based on career adaptability and career decision-making self-efficacy

**DOI:** 10.3389/fpsyg.2026.1892183

**Published:** 2026-09-17

**Authors:** Yang Yang, Jiazhu Li

**Affiliations:** 1 School of Computer and Information Technology, Xinyang Normal University, Xinyang, Henan, China; 2 School of Foreign Languages, Xinyang Normal University, Xinyang, Henan, China

**Keywords:** career adaptability, career decision-making self-efficacy, college students, employment anxiety, future time perspective

## Abstract

**Background:**

Employment anxiety among college students has become increasingly prominent under intensified employment pressure. This study examined the associations among future time perspective, career adaptability, employment anxiety, and career decision-making self-efficacy, drawing on Social Cognitive Career Theory and Career Construction Theory.

**Methods:**

A survey was conducted with 1,100 Chinese college students using standardized measures. Partial least squares structural equation modeling was used to analyze the associations among future time perspective, career adaptability, employment anxiety, and career decision-making self-efficacy.

**Results:**

Future time perspective was negatively associated with employment anxiety and positively associated with career adaptability. Career adaptability was negatively associated with employment anxiety and was involved in the association between future time perspective and employment anxiety. Career decision-making self-efficacy moderated the associations of future time perspective and career adaptability with employment anxiety, with a numerically larger interaction coefficient observed for the association between career adaptability and employment anxiety. The negative indirect association between future time perspective and employment anxiety through career adaptability was more pronounced at higher levels of career decision-making self-efficacy. Multi-group analysis showed that the negative association between career adaptability and employment anxiety was more pronounced among female students.

**Conclusion:**

The findings clarify the associations among future time perspective, career adaptability, career decision-making self-efficacy, and employment anxiety among Chinese college students. These results may provide evidence for university career counseling and psychological support practices.

## 1 Introduction

In recent years, the steady rise of China’s higher education system has produced larger graduation cohorts, producing a more competitive employment market. As a result, employment anxiety (EA) among college students has gradually spread to lower-grade cohorts (Wang et al., 2025). EA typically refers to the negative emotions such as anxiety, concern, and unease that individuals experience when facing competition, uncertainty, and potential failure risks in job-seeking scenarios (Liu and Zhang, 2025). Survey data indicates that about one-third of college students perceive significant employment pressure, and nearly half report high levels of EA (Wang and Zhang, 2025). Prolonged EA is closely linked to negative states such as sleep disorders, depressive symptoms, and reduced job-seeking motivation, and it may interfere with career decision-making and job-seeking behaviors (Liu and Zhang, 2025).

To better understand the background of EA, existing research has explored it from multiple perspectives, such as personality traits (Wan et al., 2024), family support (Shao et al., 2020), social comparison (Jin et al., 2024), and psychological capital (Zhao, 2023). Additionally, according to Social Cognitive Career Theory (SCCT; Lent et al., 1994), a key factor in coping with employment pressure lies in whether individuals possess a positive future-oriented cognitive tendency and career adaptability (CA) resources. Specifically, Future Time Perspective (FTP) helps individuals form a sense of direction and goal awareness, while CA reflects their ability to cope with changes and challenges. Together, these two factors form the core psychological foundation for college students to handle career uncertainties. Existing studies have provided empirical support for these factors. FTP, as a positive future-oriented construct, has been found to be negatively correlated with EA among college students (Nguyen and Tran, 2025). Similarly, CA, another core psychological resource, has also been shown to negatively correlate with EA, effectively alleviating students’ stress during the job-seeking process (Fang and Xu, 2025). Notably, some research suggests that the alleviating effect of CA on EA may vary by gender, with females possibly being more likely to utilize CA resources to cope with employment pressure. This potential gender difference warrants further investigation (Nuutinen et al., 2025). Additionally, SCCT emphasizes that self-efficacy serves as a crucial aspect in the professional growth process. According to this paradigm, Career Decision-Making Self-Efficacy (CDMSE) is a crucial cognitive resource that influences how people approach decisions connected to their careers and how they feel about those decisions (Manap et al., 2025).

Most existing studies have examined FTP and CA in relation to EA as separate predictors, while work that systematically investigates their joint functioning and the role of cognitive resources such as CDMSE remains scarce. Building on this gap, the present study incorporates SCCT and Career Construction Theory (CCT) to develop a framework that includes both mediation and moderation routes, aiming to clarify how future-oriented cognition connects to emotional outcomes as well as the conditions under which these associations vary.

### 1.1 Theoretical framework and hypotheses

#### 1.1.1 Theoretical basis

This study uses SCCT as the primary theoretical framework, complemented by CCT, to develop an integrated model that explains the mechanisms linking FTP to EA among college students, as well as the moderating conditions involved.

SCCT describes how personal attributes, contextual conditions, and behavioral tendencies operate together, and it underscores the importance of cognitive components such as self-efficacy and outcome expectations within the broader process of career development (Wang et al., 2022). In this study, FTP is considered an important factor reflecting an individual’s future-oriented cognition, which may be related to the development of CA resources and emotional experiences (Ling et al., 2022). SCCT also posits that self-efficacy is closely associated with career-related emotions and with how individuals engage with career-related tasks (Ran and Cinamon, 2023). In the present model, CDMSE is considered a task-specific efficacy belief whose moderating role may differ across three conceptually distinct associations. The association between FTP and EA links future orientation with employment-related emotion, the association between FTP and CA links future orientation with adaptive career resources, and the association between CA and EA links adaptive career resources with employment-related emotion. Accordingly, the theoretical rationale for CDMSE is specified separately for each association rather than assuming the same moderating rationale across all three relationships (Alromaihi et al., 2026). Therefore, SCCT provides a critical explanatory foundation for the relationships among FTP, CA, and EA, as well as the moderating role of CDMSE. Complementing SCCT, CCT offers further theoretical support for the mediation process. According to CCT, individuals adapt to external environments by actively constructing their career development trajectories, with CA being a key ability in this process (Chang et al., 2023). CCT emphasizes that future-oriented attention and planning help individuals accumulate adaptive resources, enabling them to handle challenges in career transition situations more effectively.

In conclusion, this study integrates SCCT and CCT to propose a model that incorporates both mediation and moderation structures. SCCT provides a conceptual foundation for understanding the relationship from future-oriented cognition to CA resources and emotional experiences, clearly indicating the moderating significance of CDMSE. CCT further elaborates on how future orientation is linked to the development of CA resources. Together, these theories offer a systematic and robust theoretical support for the hypotheses of this study.

### 1.2 Future time perspective and employment anxiety

FTP is typically defined as an individual’s cognition and perception of their future time, future opportunities, and the likelihood of achieving their goals. (Jin et al. (2025) suggest that individuals with a higher FTP are often able to guide their current behavior with long-term goals and maintain a higher level of psychological resources in stressful situations. During college students’ development, FTP not only influences their academic engagement and goal management but also closely relates to career planning, job preparation, and resource allocation. According to research, college students who have a more expansive viewpoint are more inclined to pursue job exploration, obtain employment information, and uphold optimistic career expectations (Cheng and Nguyen, 2023). Consistent with this emphasis on proactive career engagement, recent research among higher education students reported positive associations of career proactivity with self-regulated learning and career sustainability (Qalati et al., 2025).

There is a strong negative association between FTP and EA, according to a large body of studies. Priastanti and Pratitis (2021) found that college students with a clearer future orientation reported lower levels of anxiety when facing employment uncertainty. Jia et al. (2022) also pointed out that during career transitions, FTP negatively correlates with anxiety and concerns among young people. From the perspective of SCCT, future orientation and outcome expectations are closely linked to emotional experiences in career situations. Higher FTP is associated with clearer perceptions of future opportunities, more positive career expectations, and lower anxiety under employment pressure (Phan, 2015; Lu and Jia, 2022). Accordingly, we hypothesize that FTP is negatively associated with EA among college students. Moreover, FTP may be indirectly associated with EA through CA. According to CCT, individuals need to utilize adaptability to manage uncertainty during career transitions or role changes (Chang et al., 2023). The theory emphasizes that individuals’ attention to and planning for the future are closely tied to the development of CA resources (Diaconu-Gherasim et al., 2024). College students with a higher FTP are more likely to have a better sense of direction in their career narratives, making them more inclined to engage in job preparation activities and build adaptive resources. This process is especially crucial in high-pressure employment situations, as further supported by research. Ling et al. (2022) found that the higher the level of future orientation, the richer the CA resources among college students in career planning. At the same time, a substantial negative association occurs between CA and EA. For example, Fang and Xu (2025) noted that students with higher adaptability levels are better at managing career uncertainty, and thus report lower levels of EA. Accordingly, this study examines whether FTP is indirectly associated with EA through CA.

### 1.3 The mediating role of career adaptability

CA refers to a psychological capacity associated with regulating actions and emotions when individuals face career-related tasks, transitions, and uncertainty (Zhao et al., 2024). Among college students preparing for employment, higher CA is associated with greater flexibility in career exploration and decision-making, better stress management, and more favorable psychological adjustment during career transitions (Lee and Jung, 2022).

Evidence links higher FTP with greater CA. Boo et al. (2021) further reported that college students with higher FTP tend to show clearer goal setting and stronger career-related psychological resources. According to SCCT, future orientation can be linked to cognitive mechanisms such as self-regulation and outcome expectations, which in turn are associated with an individual’s level of adaptive resources in career contexts (Fang et al., 2024; Imbellone and Laghi, 2016). This emphasis on intermediary psychological processes is also reflected in recent career sustainability research reporting indirect associations through psychological states (Qalati et al., 2026). During the job-seeking phase, this future-oriented cognition may help students maintain goal consistency and enhance their engagement in career exploration tasks, which is accompanied by higher CA. CA is negatively associated with EA. Gao et al. (2025) reported that greater adaptation was associated with greater emotional stability under employment pressure and less fear of future uncertainty. CCT further emphasizes that CA is a crucial resource for individuals to manage stress, maintain psychological balance, and construct their professional identity during career transitions (Zhao et al., 2023). Therefore, in the context of employment challenges, higher CA may be associated with more constructive responses to difficulties, greater psychological resilience, and lower levels of anxiety. In conclusion, FTP may be indirectly associated with EA through CA. This leads us to hypothesize a significant indirect association between FTP and EA through CA.

### 1.4 The moderating role of career decision-making self-efficacy

SCCT indicates that self-efficacy plays an essential role in understanding how individuals approach career-related actions, regulate emotions, and adjust to changing circumstances (Yang et al., 2025). For college students, CDMSE represents a meaningful psychological resource that supports activities such as career exploration, information seeking, making career choices, and handling uncertainty. Empirical work suggests that students with higher CDMSE often show stronger readiness for career preparation and report fewer experiences of helplessness and anxiety (Cai et al., 2025).

First, the proposed moderation of the association between FTP and EA concerns the relationship between future orientation and employment-related emotion. FTP reflects students’ orientation toward future opportunities and goals, whereas CDMSE reflects their confidence in completing career decision-making tasks. At higher levels of CDMSE, stronger future orientation may be accompanied by greater confidence in evaluating and pursuing career-related goals (Li et al., 2025). Under this condition, the negative association between FTP and EA is expected to be more pronounced. By contrast, at lower levels of CDMSE, future orientation may coexist with greater uncertainty regarding career decision-making tasks, and the negative association between FTP and EA may therefore be less pronounced. Existing findings showing associations between career-related self-efficacy and lower levels of employment-related anxiety are consistent with this theoretical expectation (Han, 2022; Zhang et al., 2023).

Second, the proposed moderation of the association between FTP and CA concerns the relationship between future orientation and adaptive career resources. FTP provides temporal direction and goal awareness, whereas CA reflects adaptive resources relevant to career demands, transitions, and uncertainty. The FTP–CA association may be more pronounced among students who report stronger confidence in carrying out specific career decision-making activities. In particular, CDMSE encompasses self-assessment, information gathering, goal setting, planning, and problem solving, which are conceptually relevant to how students organize future goals within career contexts (Zhang et al., 2025). Earlier evidence has identified a positive relationship between FTP and CA (Ling et al., 2022), while CDMSE has also been closely associated with career preparation and CA (Lee and Jung, 2022). On this basis, the positive association between FTP and CA is expected to be more pronounced at higher levels of CDMSE.

Third, the proposed moderation of the association between CA and EA concerns the relationship between adaptive career resources and employment-related emotion. CA reflects resources associated with managing career uncertainty, whereas CDMSE reflects confidence in applying career decision-related competencies (Gao et al., 2025). At higher levels of CDMSE, greater CA may be more closely accompanied by confidence in applying adaptive strategies when students face career uncertainty (Zhang et al., 2025). Under this condition, the negative association between CA and EA is expected to be more pronounced. This explanation conceptualizes CA and CDMSE as complementary career-related psychological resources rather than interchangeable resources (Alkal, 2025). The documented association between CA and CDMSE is also consistent with this complementary-resource interpretation. Recent evidence among university graduates similarly reports positive associations between CA and both future orientation and CDMSE (Badwy et al., 2025).

### 1.5 This study

Amid increasingly challenging employment conditions, EA has emerged as a prominent concern for college students and is closely tied to their psychological well-being and career development. Although prior work has examined FTP, CA, and CDMSE as separate correlates of EA, notable gaps remain. One major gap is that much of the existing research concentrates on direct associations among these variables, with limited attention to how CA may function as a mediating mechanism between FTP and EA. This has led to an insufficient understanding of how FTP is associated with emotional outcomes through psychological resources. Second, as a core cognitive variable in SCCT, the moderating role of CDMSE in the above-mentioned associations has not been fully integrated. More specifically, limited attention has been given to whether CDMSE has distinct conditional roles in the association between FTP and EA, the association between FTP and CA, and the association between CA and EA. This distinction is particularly important because these three associations involve different combinations of future orientation, adaptive career resources, and employment-related emotion.

To address these gaps, this study makes two theoretical advancements. First, it validates and deepens the applicability of SCCT and CCT in the context of college students’ employment psychology. This research integrates SCCT and CCT to develop a cognitive-resource-emotion integration model encompassing individual cognition, psychological resources, and emotional outcomes, aiming to provide a more systematic explanation of the associations among FTP, CA, and EA in stressful employment situations. Second, it clarifies the conditions under which the association between FTP and EA varies. This study introduces CDMSE as a key contextual variable, exploring whether the indirect association between FTP and EA through CA varies across levels of CDMSE. This design not only addresses the associational question of “whether and how FTP is indirectly associated with EA through CA” but also addresses the conditional question of “at what level of self-efficacy this indirect association is more pronounced,” thereby enhancing both theoretical depth and practical application value in employment counseling.

Drawing on the above theoretical insights and existing evidence, this study seeks to clarify how FTP relates to college students’ EA and to identify the conditions under which these associations vary by adopting a moderated mediation framework. Specifically, the study examines whether the indirect association between FTP and EA through CA varies across different levels of CDMSE. Figure 1 presents the conceptual model, and the corresponding hypotheses are outlined below.

**Figure 1:**
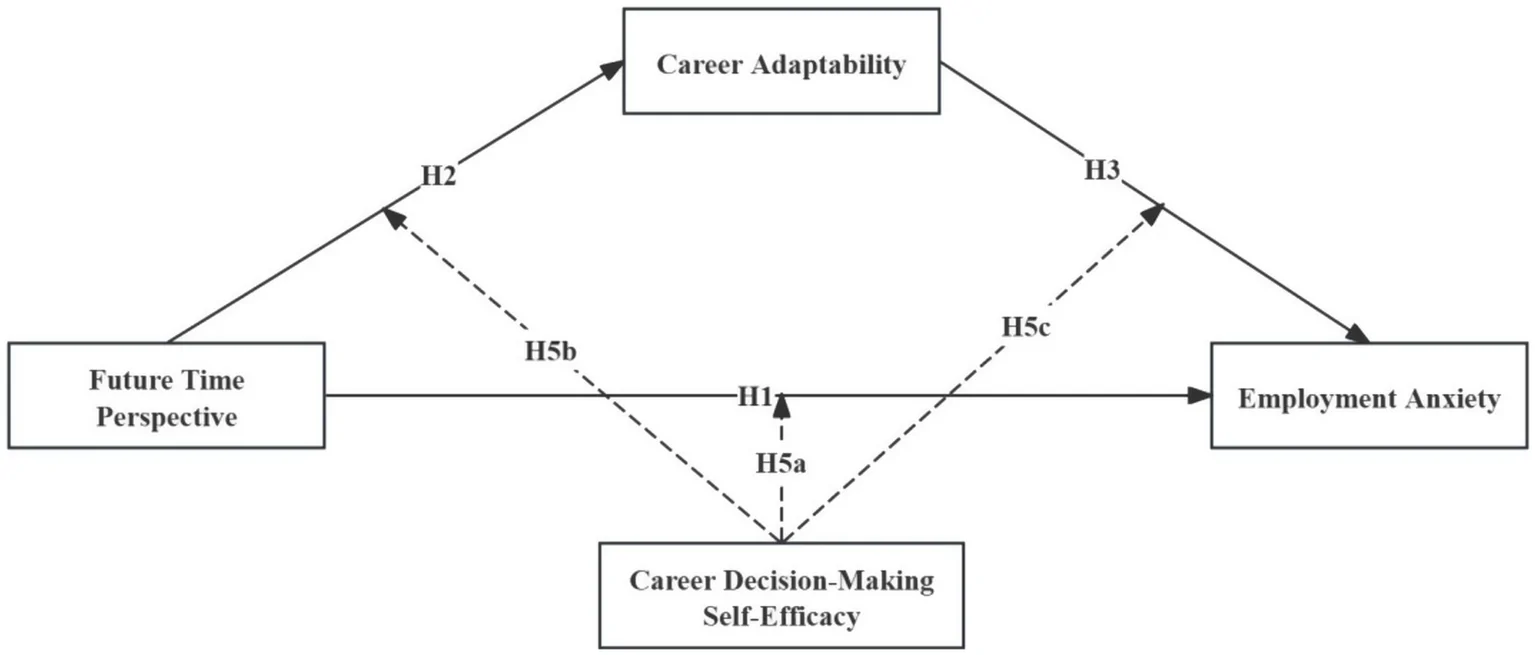
Theoretical hypothesis model.

H1: FTP is negatively associated with EA.

H2: FTP is positively associated with CA.

H3: CA is negatively associated with EA.

H4: FTP is indirectly associated with EA through CA.

H5a: CDMSE negatively moderates the relationship between FTP and EA.

H5b: CDMSE positively moderates the relationship between FTP and CA.

H5c: CDMSE negatively moderates the relationship between CA and EA.

H6: The indirect association between FTP and EA through CA varies across different levels of CDMSE.

## 2 Materials and methods

### 2.1 Participants

This study employed a cross-sectional design targeting college students. A two-stage sampling procedure was used. At the institutional level, three universities in Henan, China, were recruited through a non-probability procedure based on accessibility and willingness to participate. The participating institutions included comprehensive, science and engineering, and liberal arts universities, providing variation in institutional settings. The identities of the participating universities were kept confidential. At the student level, simple random sampling was conducted within each participating university. After obtaining approval from the academic affairs or student affairs department of each university, the research team obtained lists of eligible students across different grades and majors to serve as sampling frames. Computer-generated random numbers were then used to select the required students from each university’s complete list of eligible students. Thus, probability sampling was applied to student selection within the three participating universities rather than to the selection of universities themselves. At each participating university, the research team collaborated with faculty members to inform the selected students about the study purpose, voluntary participation, and confidentiality procedures. Students who agreed to participate completed the questionnaire independently. Data collection took place from September 2025 to November 2025, covering a stable teaching period in the new semester and avoiding exam weeks and other special periods. The surveys were distributed and collected anonymously through the Questionnaire Star platform (www.sojump.com).

The questionnaire comprised 89 items: 86 items from the four study instruments and three demographic items assessing gender, age, and academic year. An *a priori* power analysis using G*Power 3.1 was based on linear multiple regression with a maximum of eight predictors. With f^2^ = 0.02, *α* = 0.05, and power = 0.90, the minimum required sample size was 962. Of the 1,150 questionnaires returned, responses were excluded if (1) more than five items were missing; (2) at least 80% of responses were rated as “Strongly Agree” or “Strongly Disagree”; or (3) completion time was substantially shorter than typical (Bowling et al., 2023; Meade and Craig, 2012). After screening, 1,100 valid questionnaires remained, corresponding to a valid-response rate of 95.65%. The final sample exceeded the required size and included 540 male and 560 female students across all undergraduate years (Table 1).

**Table 1:** Sample characteristics (*N* = 1,100).

Variable	Group	*N*	%
Gender	Female	560	50.9
	Male	540	49.1
Age	18–19	290	26.4
	20–21	264	24.0
	22–23	292	26.5
	>24	254	23.1
Grade	Freshman	295	26.8
	Sophomore	243	22.1
	Junior	266	24.2
	Senior	296	26.9

### 2.2 Measurement tools

#### 2.2.1 Future time perspective

The FTP scale developed by Song (2004) contains 20 items across five domains: behavioral commitment, future efficacy, long term goal orientation, future purpose awareness, and future imagery. Responses are rated on a 5-point Likert scale, with five reverse-scored items; higher scores indicate stronger future orientation (Wei et al., 2025). The scale showed high internal consistency in this sample (Cronbach’s *α* = 0.939) and has been validated among Chinese college students (Ling et al., 2022).

#### 2.2.2 Employment anxiety

The Chinese Future Employment Anxiety Scale (Wang et al., 2025) comprises 17 items across four dimensions: personal ability, knowledge application, career substitutability, and social relationships. Items are rated on a 5-point Likert scale from Strongly Disagree to Strongly Agree, with higher scores indicating greater EA. The scale has been validated among Chinese college students and showed high internal consistency in this sample (Cronbach’s *α* = 0.945).

#### 2.2.3 Career adaptability

The CA scale developed by Savickas (1997) and translated into Chinese by Hou et al. (2012) comprises 24 items across four components: concern, control, curiosity, and confidence. Items are rated on a 5-point Likert scale, with higher total scores indicating greater CA. Its psychometric properties have been supported in Chinese samples (Wu and Gao, 2025), and Cronbach’s *α* was 0.947 in this study.

#### 2.2.4 Career decision-making self-efficacy

The CDMSE scale developed by Betz et al. (1996) and revised for Chinese populations by Peng and Long (2001) comprises 25 items assessing self-assessment, information gathering, goal setting, planning, and problem solving. Items are rated on a 5-point Likert scale, with higher scores indicating greater confidence in career decision-making. Its reliability and validity have been supported in Chinese research (Fang et al., 2025), and Cronbach’s *α* was 0.948 in this study.

## 3 Results

Skewness and kurtosis were within the specified limits of 3 and 10, respectively (Kim, 2013). Bartlett’s tests were significant for all scales (*p* < 0.001; Table 2).

**Table 2:** Distribution and descriptive indices (*N* = 1,100).

Constructs	MIN	MAX	M ± SD	Skew	Kurt	Bartlett
FTP	17	80	52.630 ± 11.997	−0.341	0.540	<0.001
CA	26	93	60.220 ± 13.686	−0.443	−0.163	<0.001
EA	14	70	37.250 ± 10.269	−0.659	−0.308	<0.001
CDMSE	25	100	71.790 ± 15.094	−0.468	0.552	<0.001

Skew, skewness; Kurt, kurtosis; FTP, future time perspective; EA, employment anxiety; CA, career adaptability; CDMSE, career decision-making self-efficacy. Statistics were based on the final retained items: FTP = 16, CA = 19, EA = 14, and CDMSE = 20.

### 3.1 SEM analysis

PLS-SEM was used to assess the measurement and structural models and examine the association between FTP and EA. The initial measurement model included four constructs and 86 scale items; the three demographic items were not treated as measurement indicators. FTP, CA, EA, and CDMSE were specified as first-order reflective constructs, with item-level indicators loading directly on their respective constructs. No higher-order construct specification was used. The original subdimensions of the instruments were retained as the conceptual basis for evaluating content coverage but were not modeled as separate lower-order components, because the study hypotheses focused on associations among the overall constructs. The analysis was based on data from 1,100 students, and the use of PLS-SEM followed Hair et al. (2021).

### 3.2 Measurement model

To evaluate the validity and reliability of the study scales, the measurement model was analyzed in accordance with Hair et al. (2021). Reliability was evaluated using outer loadings and Composite Reliability values. The initial measurement model contained 86 scale items. Seventeen items with outer loadings below 0.60 were removed: FTP4, FTP9, FTP12, and FTP20; CA4, CA7, CA14, CA23, and CA24; EA5, EA7, and EA13; and CDMSE1, CDMSE8, CDMSE14, CDMSE20, and CDMSE22. The final measurement model therefore contained 69 items: 16 for FTP, 19 for CA, 14 for EA, and 20 for CDMSE. After statistical screening, the retained item sets were also checked against the original domain structure of each instrument. The retained items continued to represent all five FTP domains, all four CA domains, all four EA dimensions, and all five CDMSE areas; thus, no original content domain was omitted. This domain-level review supported the preservation of the conceptual content coverage of each construct after item deletion. Cronbach’s alpha (>0.70), Composite Reliability (>0.80), and Average Variance Extracted (>0.50) met the specified thresholds (Table 3). Discriminant validity was supported by HTMT values below 0.85 and the Fornell–Larcker criterion (Tables 4, 5).

**Table 3:** Measurement quality indices (*N* = 1,100).

Constructs	Items	Loadings	Cronbach’*α*	CR	AVE
EA	EA1	0.773	0.945	0.946	0.585
	EA2	0.801			
	EA3	0.752			
	EA4	0.754			
	EA6	0.724			
	EA8	0.792			
	EA9	0.781			
	EA10	0.777			
	EA11	0.777			
	EA12	0.779			
	EA14	0.733			
	EA15	0.697			
	EA16	0.770			
	EA17	0.788			
FTP	FTP1	0.758	0.939	0.942	0.524
	FTP2	0.777			
	FTP3	0.782			
	FTP5	0.754			
	FTP6	0.628			
	FTP7	0.782			
	FTP8	0.625			
	FTP10	0.663			
	FTP11	0.804			
	FTP13	0.776			
	FTP14	0.802			
	FTP15	0.777			
	FTP16	0.676			
	FTP17	0.631			
	FTP18	0.661			
	FTP19	0.630			
CA	CA1	0.658	0.947	0.951	0.505
	CA2	0.783			
	CA3	0.647			
	CA5	0.659			
	CA6	0.753			
	CA8	0.681			
	CA9	0.688			
	CA10	0.737			
	CA11	0.653			
	CA12	0.633			
	CA13	0.703			
	CA15	0.667			
	CA16	0.726			
	CA17	0.749			
	CA18	0.745			
	CA19	0.827			
	CA20	0.658			
	CA21	0.756			
	CA22	0.739			
CDMSE	CDMSE2	0.763	0.948	0.952	0.504
	CDMSE3	0.710			
	CDMSE4	0.792			
	CDMSE5	0.727			
	CDMSE6	0.731			
	CDMSE7	0.614			
	CDMSE9	0.617			
	CDMSE10	0.610			
	CDMSE11	0.802			
	CDMSE12	0.792			
	CDMSE13	0.780			
	CDMSE15	0.800			
	CDMSE16	0.656			
	CDMSE17	0.741			
	CDMSE18	0.752			
	CDMSE19	0.617			
	CDMSE21	0.702			
	CDMSE23	0.631			
	CDMSE24	0.664			
	CDMSE25	0.629			

CR, composite reliability; AVE, average variance extracted; FTP, future time perspective; EA, employment anxiety; CA, career adaptability; CDMSE, career decision-making self-efficacy.

**Table 4:** HTMT results (*N* = 1,100).

Constructs	EA	FTP	CA	CDMSE
EA				
FTP	0.382			
CA	0.353	0.288		
CDMSE	0.189	0.253	0.191	

FTP, future time perspective; EA, employment anxiety; CA, career adaptability; CDMSE, career decision-making self-efficacy.

**Table 5:** Fornell–Larcker matrix for testing discriminant validity (*N* = 1,100).

Constructs	EA	FTP	CA	CDMSE
EA	***0.765***			
FTP	−0.359	***0.724***		
CA	−0.367	0.292	***0.710***	
CDMSE	−0.186	0.234	0.192	***0.710***

FTP, future time perspective; EA, employment anxiety; CA, career adaptability; CDMSE, career decision-making self-efficacy; The square root of the AVE value is displayed in bold and italic on the diagonal of the table.

### 3.3 Common method bias and confirmatory factor analysis

Harman’s single-factor test yielded 11 factors with eigenvalues above one, with the first accounting for 23.414% of the variance, below the 40% criterion (Podsakoff and Organ, 1986). Adding a common latent factor linked to all observed indicators did not produce a significant model difference (*p* > 0.05), providing no indication of substantial common method bias. Confirmatory factor analysis yielded *χ*^2^/df = 3.537, SRMR = 0.042, GFI = 0.912, NFI = 0.914, IFI = 0.928, TLI = 0.913, CFI = 0.935, and RMSEA = 0.045, indicating acceptable model fit according to Hu and Bentler (1998).

### 3.4 Structural model

#### 3.4.1 Collinearity test

VIF values were examined for the predictor constructs (Hair et al., 2021). All values were below 3.3, indicating no substantial collinearity concerns (Table 6).

**Table 6:** VIF results (*N* = 1,100).

Constructs	EA	FTP	CA	CDMSE
EA				
FTP	1.141		1.063	
CA	1.114			
CDMSE	1.085		1.058	

FTP, future time perspective; EA, employment anxiety; CA, career adaptability; CDMSE, career decision-making self-efficacy.

#### 3.4.2 Significance test of structural model relationships

PLS bootstrapping with 5,000 resamples was applied to test the significance of the structural paths. All structural paths reported in Table 7 were statistically significant, with *t* values greater than 1.96, *p* values below 0.05, and confidence intervals excluding zero. The path coefficients and corresponding statistical results are shown in Table 7.

**Table 7:** Significance results of structural model relationships (*N* = 1,100).

Relationships	Original sample (O)	2.50%	97.50%	*t*	*p*
FTP → EA	−0.247	−0.314	−0.178	7.106	<0.001
FTP → CA	0.258	0.184	0.329	6.990	<0.001
CA → EA	−0.272	−0.343	−0.206	7.735	<0.001
CDMSE → EA	−0.085	−0.146	−0.023	2.653	0.008
CDMSE → CA	0.132	0.061	0.204	3.594	<0.001

FTP, future time perspective; EA, employment anxiety; CA, career adaptability; CDMSE, career decision-making self-efficacy.

Regarding explanatory power, the *R*^2^ value for EA was 0.303, and the adjusted *R*^2^ was 0.302, indicating stable explanatory performance. For CA, the *R*^2^ value was 0.327, and the adjusted *R*^2^ was 0.325, showing consistent variance explanation after adjustment. Predictive relevance was assessed using the *Q*^2^ statistic. EA recorded a *Q*^2^ value of 0.217, while CA recorded 0.272. Both values were greater than zero, suggesting acceptable predictive relevance for the two constructs. Overall, the model demonstrated adequate explanatory and predictive capability for EA and CA (see Table 8).

**Table 8:** Explanatory power of the model (*N* = 1,100).

Constructs	*R* ^2^	*R*^2^ adjusted	*Q* ^2^
EA	0.303	0.302	0.217
CA	0.327	0.325	0.272

FTP, future time perspective; EA, employment anxiety; CA, career adaptability; CDMSE, career decision-making self-efficacy.

#### 3.4.3 Mediation effect analysis

The mediating role of CA between FTP and EA was examined using PLS SEM. Gender, age, and academic year were included as control variables. The analysis showed that CA served as a partial mediator in the association between FTP and EA. Detailed mediation results are presented in Table 9.

**Table 9:** Indirect association results (*N* = 1,100).

Path	Indirect effect	2.50%	97.50%	*t*	*p*	Results
FTP → CA → EA	−0.070	−0.099	−0.047	5.175	<0.001	PM

FTP, future time perspective; EA, employment anxiety; CA, career adaptability; CDMSE, career decision-making self-efficacy; PM, partial mediation.

#### 3.4.4 Moderation effect analysis

To investigate the moderating role of CDMSE in the model, interaction terms were introduced for analysis. After adjusting for gender, age, and academic year, the results showed that the interaction between CDMSE and FTP was significantly and negatively associated with EA, and the interaction between CDMSE and CA also showed a significant negative association with EA. However, the interaction between CDMSE and FTP was not significantly associated with CA, indicating that moderation was not supported for this association. A further comparison of the absolute interaction coefficients indicated that the moderation of the association between FTP and EA (|*β*| = 0.085) was slightly weaker than that of the association between CA and EA (|*β*| = 0.106). This pattern suggests that the magnitude of the moderating association of CDMSE differed across the tested relationships, with a larger interaction coefficient for the association between CA and EA. Table 10 presents the detailed moderation results.

**Table 10:** Moderation results (*N* = 1,100).

Relationships	Original sample (O)	2.50%	97.50%	*t*	*p*	Results
CDMSE × FTP → EA	−0.085	−0.149	−0.024	2.646	0.008	Yes
CDMSE × FTP → CA	0.032	−0.025	0.086	1.122	0.262	No
CDMSE × CA → EA	−0.106	−0.176	−0.035	2.924	0.003	Yes

FTP, future time perspective; EA, employment anxiety; CA, career adaptability; CDMSE, career decision-making self-efficacy.

#### 3.4.5 Moderated mediation analysis

Using 5,000 bootstrap resamples, the conditional indirect association between FTP and EA through CA was estimated at low (−1 SD), mean, and high (+1 SD) levels of CDMSE. The estimates were −0.037, −0.070, and −0.110, respectively, with all 95% bias-corrected confidence intervals excluding zero. The negative indirect association was more pronounced at higher levels of CDMSE, and the High vs. Low contrast was significant, with its 95% bias-corrected bootstrap confidence interval excluding zero, supporting H6 (Table 11). Figure 2 presents the overall structural model.

**Table 11:** Conditional indirect associations between FTP and EA through CA across levels of CDMSE (*N* = 1,100).

Path	CDMSE Level / Contrast	Estimate	2.50%	97.50%
FTP → CA → EA	Low (−1 SD)	−0.037	−0.073	−0.013
	Mean	−0.070	−0.098	−0.047
	High (+1 SD)	−0.110	−0.161	−0.070
Contrast	High vs. Low	−0.072	−0.130	−0.021

FTP, future time perspective; EA, employment anxiety; CA, career adaptability; CDMSE, career decision-making self-efficacy. The first three rows report conditional indirect associations. The High vs. Low row reports the contrast between the conditional indirect associations at high and low levels of CDMSE. Confidence intervals are bias-corrected bootstrap confidence intervals based on 5,000 resamples.

**Figure 2:**
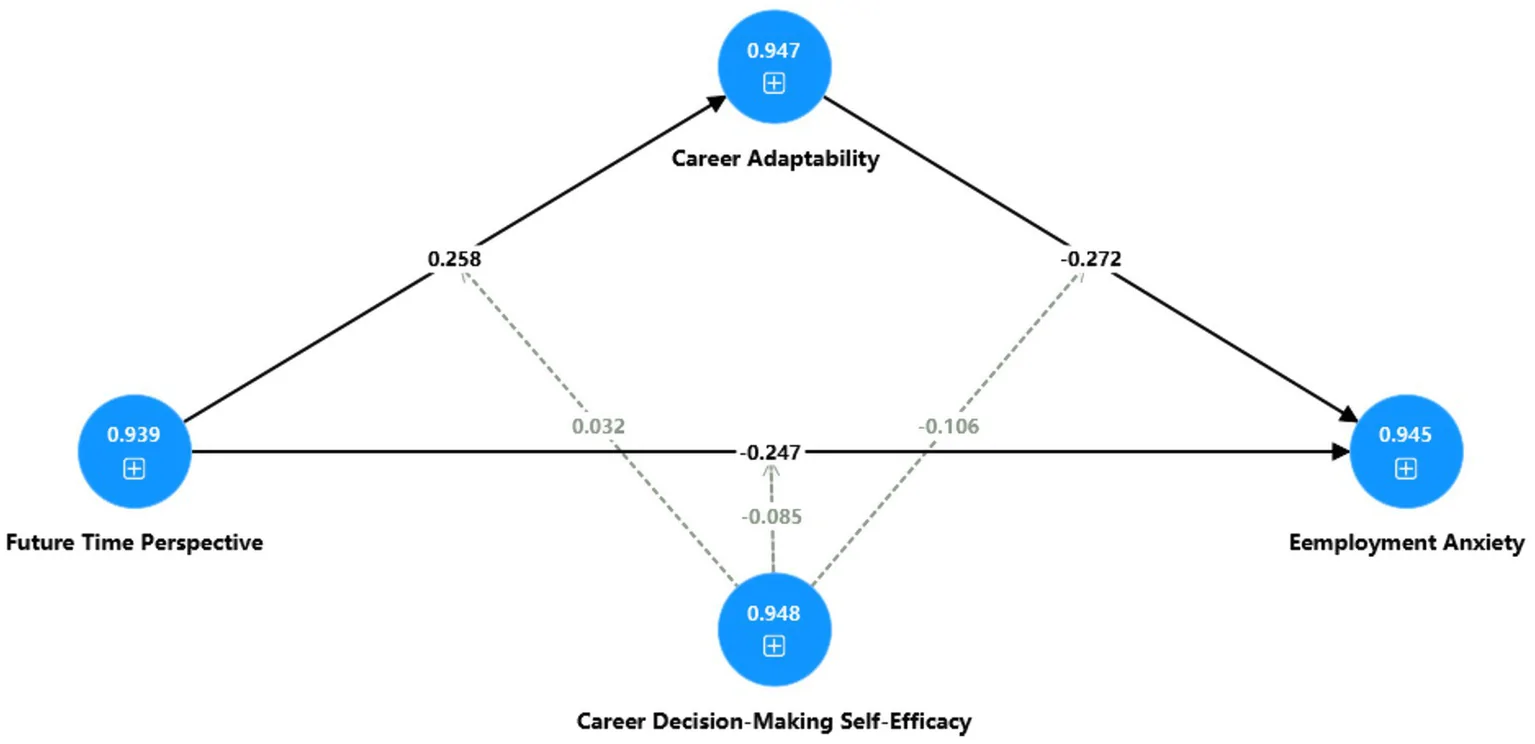
Path diagram for mediation and moderation effects. Blue circles show Cronbach’s alpha coefficients; values on the paths represent path coefficients.

### 3.5 Multi-group analysis

#### 3.5.1 Measurement invariance analysis

Using the MICOM method suggested by Henseler et al. (2016), measurement invariance was investigated. As presented in Table 12, compositional invariance was established for EA, CA, and CDMSE, whereas FTP did not meet the compositional invariance criterion. Accordingly, group comparisons involving FTP were not interpreted in the subsequent MGA.

**Table 12:** MICOM step 2: Compositional invariance by gender (*N* = 1,100).

Constructs	Correlation permutation mean	5% quantile of empirical distribution of *c_u_*	*p*	Compositional invariance?
EA	1.000	0.999	0.197	Yes
FTP	0.998	0.994	0.034	No
CA	0.997	0.993	0.124	Yes
CDMSE	0.992	0.974	0.113	Yes

FTP, future time perspective; EA, employment anxiety; CA, career adaptability; CDMSE, career decision-making self-efficacy.

#### 3.5.2 Multi-group analysis

A gender-based PLS-MGA was conducted by dividing participants into female and male groups and comparing the group-specific coefficients (Cheah et al., 2023). Given the MICOM results, group comparisons involving FTP were not interpreted because compositional invariance was not established for FTP. As shown in Table 13, the association between CA and EA differed significantly between the female and male groups (*p* = 0.028). The negative association between CA and EA was more pronounced in the female group (*β* = −0.361) than in the male group (*β* = −0.217).

**Table 13:** Path coefficient differences by gender (*N* = 1,100).

Path	*β* (female)	*β* (male)	Δ*β* (female − male)	2.50%	97.50%	*p*
CA → EA	−0.361	−0.217	−0.144	−0.306	−0.115	0.028

FTP, future time perspective; EA, employment anxiety; CA, career adaptability.

## 4 Discussion

### 4.1 Future time perspective and employment anxiety

This study found a significant negative association between FTP and EA among college students (*β* = −0.247), with higher FTP associated with lower EA. This result supports H1. The finding is consistent with several recent studies. Nguyen and Tran (2025) suggested that FTP, as a positive cognitive motivational variable, is associated with how individuals organize distant career goals into more concrete tasks at the present stage and with lower levels of negative emotional responses to future uncertainty. According to SCCT, an individual’s cognitive representation of the future is a key personal factor related to emotional experiences in career development (Yang et al., 2025). A positive, future-oriented cognitive framework is associated with greater confidence in achieving career goals and with lower perceived threat, helplessness, and anxiety during job searching (Imbellone and Laghi, 2016). In terms of cultural context, the high emphasis on success in Chinese culture, coupled with widespread concerns about future uncertainty and competition, may be associated with higher levels of EA among college students (Zhang et al., 2025; Tu and Liu, 2025). In this cultural atmosphere, college students are more likely to perceive future uncertainty as a threat to personal success and to report higher levels of anxiety. Nevertheless, the present findings also show some cross-cultural consistency. Research involving undergraduate students in China and the United States has similarly reported negative associations between FTP and career anxiety (Boo et al., 2021), while evidence from Vietnamese undergraduates has also shown a negative association between FTP and career anxiety (Nguyen and Tran, 2025). Taken together, these parallels suggest that the negative association between FTP and career-related anxiety may extend beyond the Chinese context. However, because the present study did not directly compare cultural groups, further cross-cultural research is needed to determine whether the strength or meaning of this association differs across cultural settings.

### 4.2 The mediating role of career adaptability

The study revealed a significant positive association between FTP and CA (*β* = 0.258), supporting H2. Specifically, higher FTP has been associated with stronger tendencies to link future goals with present actions through career planning and resource preparation, alongside higher CA (Jia et al., 2022). Boo, Wang (Boo et al., 2021) further reported that students with an optimistic view of future opportunities tend to demonstrate stronger career control and confidence when dealing with career uncertainty, which is also associated with greater readiness to navigate career challenges. Additionally, CA was found to have a significant negative association with EA (*β* = −0.272), supporting H3. Gao et al. (2025) highlighted that higher levels of CA are associated with more favorable mental health and lower EA in the context of employment-related stress and uncertainty. This result aligns with CCT, which conceptualizes adaptive resources as closely related to adjustment during career-related tasks and transitions (Chang et al., 2023). Mediation analysis further revealed a significant indirect association between FTP and EA through CA (indirect effect = −0.070), confirming H4. Recent research has reported similar indirect associations. For instance, previous research reported an indirect association between future work self and career decision-making challenges through CA, highlighting the relevance of adaptive resources to future-oriented career cognition (Jia et al., 2022). This result aligns with the core idea of SCCT, which conceptualizes personal cognition, self-regulatory resources, and emotional outcomes as interrelated in career contexts (Boo et al., 2021). In this study, FTP, as an important personal cognitive variable, was associated with students’ expectations and plans for their career futures, while CA represented practical resources related to managing career development tasks. The significant indirect association indicates that these constructs are meaningfully related, but the cross-sectional design does not establish a temporal or causal sequence among them.

### 4.3 The moderating role of career decision-making self-efficacy

This study further found that CDMSE significantly negatively moderates the relationship in two paths: first, between FTP and EA (H5a is supported), and second, between CA and EA (H5c is supported). However, the moderating effect of CDMSE between FTP and CA was not significant (H5b is not supported). The specific analysis is as follows.

First, CDMSE significantly moderated the association between FTP and EA (*β* = −0.085), supporting H5a. The negative association between FTP and EA was more pronounced at higher levels of CDMSE. This pattern is consistent with the conceptual distinction between future orientation and confidence in completing career decision-making tasks. Recent evidence among university students has also shown that FTP is positively associated with career decision-making self-efficacy (Li et al., 2025). Students reporting both stronger future orientation and higher CDMSE may also report a more favorable combination of future career expectations, confidence in career decisions, and employment-related emotional experiences (Li et al., 2023). By contrast, lower CDMSE has been associated with greater career decision-making difficulties (Bi et al., 2023). Accordingly, when CDMSE is lower, uncertainty regarding career decision-making tasks may coexist with stronger future orientation, and the negative association between FTP and EA may therefore be less pronounced. This interpretation is consistent with previous evidence linking self-efficacy with employment-related emotional experiences among college students (Zhang et al., 2023). Second, CDMSE also moderated the association between CA and EA (*β* = −0.106). This result supports H5c, which is consistent with the view in SCCT. Su et al. (2024) suggest that CDMSE is a crucial belief system for individuals in coping with career challenges. The results indicate that the negative association between CA and EA is more pronounced at higher levels of CDMSE. This pattern is consistent with viewing CA and CDMSE as distinct but jointly relevant psychological resources, such that stronger career decision-making confidence is associated with a stronger negative association between CA and EA. The finding therefore provides further evidence that the association between career-related psychological resources and EA may vary according to the combination of resources available to students.

CDMSE did not significantly moderate the association between FTP and CA (*β* = 0.032), so H5b was not supported. Although both FTP (Diaconu-Gherasim et al., 2024) and CDMSE (Lee and Jung, 2022) were positively associated with CA, the association between FTP and CA remained similar across different levels of CDMSE. From an SCCT perspective, this pattern may reflect the task-specific nature of self-efficacy: CDMSE reflects confidence in career decision-making tasks, whereas CA represents broader adaptive resources (Lent et al., 1994; Wang et al., 2022). Thus, CDMSE may be positively associated with CA without necessarily strengthening the association between FTP and CA, while variation in this relationship may also be associated with learning experiences or contextual conditions. Future longitudinal research could further examine whether CDMSE shows a clearer conditional role when future orientation is considered together with specific career exploration or planning behaviors.

Comparing the intensity of the moderating effects, CDMSE’s moderating effect on the path from FTP to EA was slightly weaker than its effect on the path from CA to EA. This difference suggests that CDMSE plays a relatively stronger moderating role in the association between CA and EA. One possible interpretation is that both CDMSE and CA are core resources in career development, and their functions are more closely related, which may be associated with a more pronounced interaction between them (Xu et al., 2025). Future research could explore the joint patterns of these two resources in different cultural contexts, especially in highly competitive cultural environments like China, to understand whether and how different types of psychological resources complement or substitute each other, and how such patterns are associated with college students’ career development paths and mental health.

The moderated mediation analysis further showed that the indirect association between FTP and EA through CA varied across levels of CDMSE. Specifically, the negative indirect association was more pronounced across low, mean, and high levels of CDMSE (−0.037, −0.070, and −0.110, respectively). This pattern is consistent with the significant moderation of the association between CA and EA by CDMSE, suggesting that the indirect association linking FTP, CA, and EA differs according to students’ levels of CDMSE. These findings further highlight the relevance of considering multiple psychological resources simultaneously when examining the associations among future-oriented cognition, career-related resources, and employment-related emotional experiences (Jia et al., 2022; Alkal, 2025).

### 4.4 Gender differences

The multi-group analysis showed that the negative association between CA and EA was significantly more pronounced among female college students (*β* = −0.361) than among male college students (*β* = −0.217). This finding indicates that the strength of the association between CA and EA differs across gender groups. Some researchers have pointed out that, in traditional Chinese culture, female job seekers may draw more strongly on psychological capital when coping with workplace challenges (Koekemoer et al., 2023; Zhu, 2023). This finding is consistent with the results of this study. In the career development process, higher CA among women may be more strongly associated with lower EA, especially when facing career uncertainty. Additionally, some studies have indicated that women may differ in how they mobilize CA resources and relate adaptability to emotional regulation (Yılmaz and Çıtak, 2025; Wan Othman et al., 2024). From a cultural perspective, the expectations surrounding gender roles in Chinese culture may be relevant to college students’ career development processes. Traditional culture places additional expectations on women regarding stability and adaptability in employment, and this socialization process has been proposed as one possible contextual explanation for greater attention to career preparation and coping resources among women, which may be associated with a stronger relationship between CA and EA (Huang et al., 2025; Cao et al., 2025). On the other hand, men are expected to demonstrate independence and self-reliance according to social norms, and such expectations may be associated with a lower tendency to view CA as a psychological regulation resource, which could partly correspond to the weaker association between CA and EA observed among male students (Mendieta-Izquierdo and Cuevas-Silva, 2024; Üzümçeker, 2025). However, because gender-role beliefs and socialization processes were not directly measured in this study, these interpretations should be considered tentative rather than established mechanisms.

## 5 Implications

### 5.1 Theoretical implications

First, this study integrates SCCT and CCT to build an explanatory framework centered on cognition, resources, and emotions, further strengthening the theoretical position of FTP in college students’ employment psychology. The findings extend this integrated perspective by showing that FTP is associated with EA both directly and indirectly through CA, while CDMSE conditions some, but not all, of these associations. This pattern clarifies the complementary explanatory roles of SCCT and CCT within the same model rather than treating the two frameworks as overlapping accounts.

Second, this study reveals the boundary role of CDMSE in the model, providing more specific evidence for the role of self-efficacy in SCCT. The results show that CDMSE moderated the associations between FTP and EA and between CA and EA, but not the association between FTP and CA. This differentiated pattern suggests that the conditional role of self-efficacy in SCCT may be relationship-specific rather than uniform across career-related associations.

Finally, through multi-group analysis, this study finds gender differences in the CA path, introducing a more culturally and socially nuanced perspective to career development theories. The results indicate that the association between CA and EA may vary across gender groups, but they do not establish a specific gender-role mechanism. This group variation highlights the importance of considering demographic and contextual heterogeneity when evaluating the applicability of career theories among Chinese college students.

### 5.2 Practical implications

First, at the individual student level, college students should actively cultivate their FTP. This study confirms that FTP is significantly negatively correlated with EA, suggesting that setting clear long-term career goals and breaking them down into actionable short-term tasks may be associated with lower EA and a greater sense of control over the future. Additionally, given the indirect association between FTP and EA through CA, students should engage in career exploration activities such as internships and industry interviews to gain experiences relevant to CA and coping with employment pressures.

Second, at the institutional level, given the indirect association between FTP and EA through CA and the moderating role of CDMSE, universities should normalize career planning education by offering courses like “Future Planning,” providing career interest assessments, and conducting job-seeking skills training. Career counseling centers should offer individual consultations, mock interviews, and resume coaching to support students’ CDMSE and employment-related emotional adjustment. Furthermore, to provide students with more opportunities related to career adaptability, universities should strengthen collaborations with companies, establish internship bases, and develop mentor systems, allowing students to accumulate career experience and gain more psychological resources during the employment transition period.

## 6 Study limitations and research directions

Several limitations should be considered when interpreting the associations among FTP, CA, EA, and the moderating role of CDMSE. As all focal variables were measured at a single time point, their temporal ordering and the directionality of the observed associations cannot be determined. A cross-sectional design also provides limited insight into changes in students’ experiences during the job-search process. Future research may use longitudinal or experimental designs to examine changes in psychological resources and anxiety over time. In addition, all variables were assessed through self-reports, which may involve social desirability or response biases. Behavioral indicators or interview data could provide complementary evidence. The sample was drawn from three universities in Henan, China, and the participating universities were selected through a non-probability procedure. Therefore, institutional representativeness may be limited, and caution is warranted when generalizing the findings beyond the participating universities and Henan. Future studies could include universities from different geographic regions and adopt probability-based institutional sampling to assess the broader applicability of the findings.

## 7 Conclusion

This study examined the relationships among FTP, CA, EA, and the moderating role of CDMSE within an integrated SCCT and CCT framework. The results showed that FTP was positively associated with CA and negatively associated with EA. CA was negatively related to EA, and a significant indirect association between FTP and EA through CA was observed. CDMSE moderated the associations between FTP and EA and between CA and EA, whereas its moderation of the association between FTP and CA was not statistically significant. The indirect association between FTP and EA through CA also varied across levels of CDMSE and was more pronounced at higher levels of CDMSE. Multi-group analysis indicated that the negative association between CA and EA was stronger among female students. Overall, the findings offer empirical support for considering FTP, CA, and CDMSE in university career and psychological support programs.

## Data Availability

The raw data supporting the conclusions of this article will be made available by the authors, without undue reservation.
